# Triethyl­ammonium 3,4-dihy­droxy­benzoate

**DOI:** 10.1107/S1600536810044764

**Published:** 2010-11-06

**Authors:** Li-Cai Zhu

**Affiliations:** a School of Chemistry and Environment, South China Normal University, Guangzhou 510631, People’s Republic of China

## Abstract

In the title compound, C_6_H_16_N^+^·C_7_H_5_O_4_^−^, the hy­droxy groups of the 3,4-dihy­droxy­benzoate anion form O—H⋯O hydrogen bonds to the carboxyl­ate groups of two adjacent anions, generating layers propagating in the *ac* plane. The triethyl­ammonium cations lie between these layers, forming N—H⋯O hydrogen bonds to the carboxyl­ate groups of the anions. The structure is consolidated by weak inter­molecular C—H⋯O inter­actions.

## Related literature

For the pharmacological activity of protocatechuic acid, see: Guan *et al.* (2006 ▶); Lin *et al.* (2009 ▶); Yip *et al.* (2006 ▶). For related structures, see: Li *et al.* (2007 ▶); Mazurek *et al.* (2007 ▶).
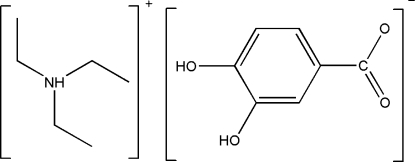


## Experimental

### 

#### Crystal data

C_6_H_16_N^+^·C_7_H_5_O_4_^−^
*M* *_r_* = 255.31
Orthorhombic, 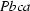

*a* = 12.4341 (16) Å
*b* = 13.7227 (18) Å
*c* = 16.150 (2) Å
*V* = 2755.7 (6) Å^3^
*Z* = 8
Mo *K*α radiation
μ = 0.09 mm^−1^
*T* = 296 K
0.32 × 0.28 × 0.28 mm

#### Data collection

Bruker APEXII area-detector diffractometer
13215 measured reflections
2483 independent reflections
1981 reflections with *I* > 2σ(*I*)
*R*_int_ = 0.028

#### Refinement

*R*[*F* ^2^ > 2σ(*F* ^2^)] = 0.037
*wR*(*F* ^2^) = 0.107
*S* = 1.03
2483 reflections
172 parameters
1 restraint
H atoms treated by a mixture of independent and constrained refinement
Δρ_max_ = 0.18 e Å^−3^
Δρ_min_ = −0.13 e Å^−3^

### 

Data collection: *APEX2* (Bruker, 2004 ▶); cell refinement: *SAINT* (Bruker, 2004 ▶); data reduction: *SAINT*; program(s) used to solve structure: *SHELXS97* (Sheldrick, 2008 ▶); program(s) used to refine structure: *SHELXL97* (Sheldrick, 2008 ▶); molecular graphics: *SHELXTL* (Sheldrick, 2008 ▶); software used to prepare material for publication: *SHELXL97*.

## Supplementary Material

Crystal structure: contains datablocks I, global. DOI: 10.1107/S1600536810044764/pv2348sup1.cif

Structure factors: contains datablocks I. DOI: 10.1107/S1600536810044764/pv2348Isup2.hkl

Additional supplementary materials:  crystallographic information; 3D view; checkCIF report

## Figures and Tables

**Table 1:** Hydrogen-bond geometry (Å, °)

*D*—H⋯*A*	*D*—H	H⋯*A*	*D*⋯*A*	*D*—H⋯*A*
O3—H3⋯O2^i^	0.82	1.85	2.6574 (14)	166
O4—H4⋯O1^ii^	0.82	1.81	2.6321 (15)	178
C7—H7⋯O1^ii^	0.93	2.57	3.235 (2)	128
N1—H12⋯O2^iii^	0.92	1.87	2.776 (2)	170
C1—H1*B*⋯O3^iv^	0.97	2.57	3.409 (2)	145
C3—H3*A*⋯O1^v^	0.97	2.55	3.516 (2)	177
C3—H3*B*⋯O3^vi^	0.97	2.56	3.351 (2)	139
C10—H10⋯O4^vii^	0.93	2.38	3.222 (2)	150

Symmetry codes: (i) 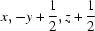; (ii) 
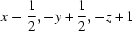
; (iii) 
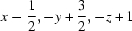
; (iv) 
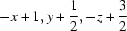
; (v) 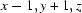; (vi) 
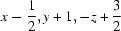
; (vii) 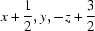.

**Fig. 1.**
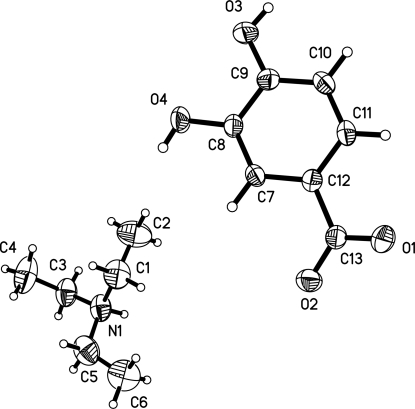
The molecular structure showing the atomic-numbering scheme and displacement ellipsoids drawn at the 50% probability level.

**Fig. 2.**
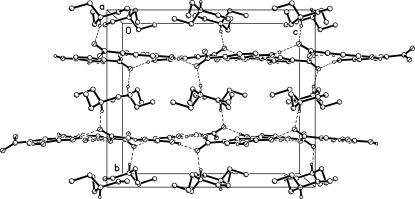
The molecular packing showing the intermolecular hydrogen bonding interactions as dashed lines.

## supplementary crystallographic information

### Comment

Protocatechuic acid (3,4-dihydroxybenzoic acid) is one of the main secondary metabolites in the plant kingdom (Guan *et al.*, 2006). Its derivatives possess diverse pharmacological activities (Lin *et al.*, 2009; Yip *et al.*, 2006). The molecular and crystal structure of the title compound is presented in this article.

The asymmetric unit of the title compound contains a 3,4-dihydroxybenzoate anion and a triethylammonium cation (Fig. 1). The bond distances and angles in the title compound sgree with the corresponding bond distances and angles reported in related structures (Li *et al.*, 2007; Mazurek *et al.*, 2007). The carboxylate group O1/O2/C13 is oriented with respect to the benzene ring at 23.18 (6)°. The hydroxy groups of the anion form O—H···O hydrogen bonds to the carboxylate groups of two other anions (Table 1), generating two-dimensional layers. The triethylammonium cations lie between these layers, forming N—H···O hydrogen bonds to the carboxylate groups of the anions (Fig. 2). The structure is  further consolidated by weak intermolecular interactions of the type C—H···O. (Table 1).

### Experimental

A mixture of protocatechuic acid (0.31 g, 2 mmol) and triethylamine (0.28 ml, 2 mmol) was stirred in methanol (20 ml) for 0.5 h at room temperature. After several days colourless block-like crystals, suitable for X-ray diffraction analysis, were obtained by slow evaporation of the solution.

### Refinement

H_12_ atom of triethylammonium cation was found from difference Fourier maps and refined isotropically with a restraint of N—H = 0.89 Å and *U*_iso_(H) = 1.5*U*_eq_(N). All other H atoms were positioned geometrically and refined as riding, with O—H = 0.82 Å and C—H = 0.93, 0.96 or 0.97 Å, for aryl, methyl and methylene type H atoms, respectively, with *U*_iso_(H) = 1.2 or 1.5*U*_eq_(C, O).

### Crystal data

**Table:**

C_6_H_16_N^+^·C_7_H_5_O_4_^−^	*F*(000) = 1104
*M**_r_* = 255.31	*D*_x_ = 1.231 Mg m^−^^3^
Orthorhombic, *P**b**c**a*	Mo *K*α radiation, λ = 0.71073 Å
Hall symbol: -P 2ac 2ab	Cell parameters from 4281 reflections
*a* = 12.4341 (16) Å	θ = 2.5–27.2°
*b* = 13.7227 (18) Å	µ = 0.09 mm^−^^1^
*c* = 16.150 (2) Å	*T* = 296 K
*V* = 2755.7 (6) Å^3^	Block, colourless
*Z* = 8	0.32 × 0.28 × 0.28 mm

### Data collection

**Table:**

Bruker APEXII area-detector diffractometer	1981 reflections with *I* > 2σ(*I*)
Radiation source: fine-focus sealed tube	*R*_int_ = 0.028
graphite	θ_max_ = 25.2°, θ_min_ = 2.5°
φ and ω scans	*h* = −14→14
13215 measured reflections	*k* = −14→16
2483 independent reflections	*l* = −16→19

### Refinement

**Table:**

Refinement on *F*^2^	Secondary atom site location: difference Fourier map
Least-squares matrix: full	Hydrogen site location: inferred from neighbouring sites
*R*[*F*^2^ > 2σ(*F*^2^)] = 0.037	H atoms treated by a mixture of independent and constrained refinement
*wR*(*F*^2^) = 0.107	*w* = 1/[σ^2^(*F*_o_^2^) + (0.0515*P*)^2^ + 0.7195*P*] where *P* = (*F*_o_^2^ + 2*F*_c_^2^)/3
*S* = 1.03	(Δ/σ)_max_ = 0.001
2483 reflections	Δρ_max_ = 0.18 e Å^−^^3^
172 parameters	Δρ_min_ = −0.13 e Å^−^^3^
1 restraint	Extinction correction: *SHELXL97* (Sheldrick, 2008), Fc^*^=kFc[1+0.001xFc^2^λ^3^/sin(2θ)]^-1/4^
Primary atom site location: structure-invariant direct methods	Extinction coefficient: 0.0034 (5)

### Special details

**Table:**

Geometry. All e.s.d.'s (except the e.s.d. in the dihedral angle between two l.s. planes) are estimated using the full covariance matrix. The cell e.s.d.'s are taken into account individually in the estimation of e.s.d.'s in distances, angles and torsion angles; correlations between e.s.d.'s in cell parameters are only used when they are defined by crystal symmetry. An approximate (isotropic) treatment of cell e.s.d.'s is used for estimating e.s.d.'s involving l.s. planes.
Refinement. Refinement of *F*^2^ against ALL reflections. The weighted *R*-factor *wR* and goodness of fit *S* are based on *F*^2^, conventional *R*-factors *R* are based on *F*, with *F* set to zero for negative *F*^2^. The threshold expression of *F*^2^ > σ(*F*^2^) is used only for calculating *R*-factors(gt) *etc*. and is not relevant to the choice of reflections for refinement. *R*-factors based on *F*^2^ are statistically about twice as large as those based on *F*, and *R*- factors based on ALL data will be even larger.

### Fractional atomic coordinates and isotropic or equivalent isotropic displacement parameters (Å^2^)

**Table:**

	*x*	*y*	*z*	*U*_iso_*/*U*_eq_	
C6	0.3472 (2)	0.97892 (16)	0.41436 (15)	0.0812 (7)	
H6A	0.3882	1.0381	0.4166	0.122*	
H6B	0.3285	0.9650	0.3579	0.122*	
H6C	0.3893	0.9263	0.4364	0.122*	
C1	0.32649 (16)	0.94605 (13)	0.60034 (13)	0.0677 (6)	
H1A	0.3919	0.9299	0.5706	0.081*	
H1B	0.2828	0.8876	0.6034	0.081*	
C2	0.3554 (2)	0.97807 (17)	0.68702 (15)	0.0893 (7)	
H2A	0.3989	1.0358	0.6844	0.134*	
H2B	0.3948	0.9271	0.7142	0.134*	
H2C	0.2908	0.9917	0.7175	0.134*	
C3	0.16443 (13)	1.05597 (13)	0.59298 (13)	0.0589 (5)	
H3A	0.1310	1.1040	0.5572	0.071*	
H3B	0.1820	1.0881	0.6448	0.071*	
C5	0.24679 (17)	0.99035 (14)	0.46451 (14)	0.0679 (6)	
H5A	0.2086	0.9287	0.4650	0.081*	
H5B	0.2008	1.0381	0.4378	0.081*	
C4	0.08368 (18)	0.97565 (17)	0.61030 (18)	0.0940 (8)	
H4A	0.0698	0.9401	0.5602	0.141*	
H4B	0.0179	1.0038	0.6302	0.141*	
H4C	0.1123	0.9322	0.6514	0.141*	
C7	0.79526 (10)	0.26562 (9)	0.58103 (8)	0.0290 (3)	
H7	0.7503	0.2736	0.5355	0.035*	
C12	0.90639 (10)	0.26034 (9)	0.56871 (8)	0.0279 (3)	
C13	0.95215 (10)	0.27035 (9)	0.48341 (8)	0.0305 (3)	
C10	0.92903 (11)	0.24288 (10)	0.71567 (9)	0.0365 (3)	
H10	0.9743	0.2354	0.7611	0.044*	
C9	0.81903 (11)	0.24855 (10)	0.72768 (8)	0.0323 (3)	
C8	0.75071 (10)	0.25922 (10)	0.65907 (8)	0.0303 (3)	
C11	0.97267 (11)	0.24818 (10)	0.63692 (9)	0.0342 (3)	
H11	1.0467	0.2436	0.6298	0.041*	
O1	1.04380 (8)	0.23683 (9)	0.47008 (7)	0.0485 (3)	
O2	0.89549 (8)	0.31388 (7)	0.42940 (6)	0.0395 (3)	
O3	0.77263 (8)	0.24370 (8)	0.80386 (6)	0.0445 (3)	
H3	0.8186	0.2309	0.8386	0.067*	
O4	0.64348 (8)	0.26312 (9)	0.67366 (6)	0.0479 (3)	
H4	0.6110	0.2621	0.6295	0.072*	
N1	0.26672 (11)	1.02205 (10)	0.55323 (10)	0.0520 (4)	
H12	0.3117 (15)	1.0749 (13)	0.5523 (12)	0.078*	

### Atomic displacement parameters (Å^2^)

**Table:**

	*U*^11^	*U*^22^	*U*^33^	*U*^12^	*U*^13^	*U*^23^
C6	0.0982 (17)	0.0615 (13)	0.0840 (15)	0.0088 (12)	0.0014 (13)	−0.0160 (11)
C1	0.0641 (12)	0.0484 (10)	0.0906 (15)	0.0088 (9)	−0.0025 (11)	0.0114 (10)
C2	0.113 (2)	0.0760 (15)	0.0786 (15)	0.0189 (14)	−0.0120 (14)	0.0185 (12)
C3	0.0446 (9)	0.0473 (10)	0.0848 (14)	0.0007 (8)	0.0011 (9)	0.0004 (9)
C5	0.0733 (13)	0.0496 (10)	0.0807 (14)	−0.0091 (9)	−0.0151 (11)	−0.0100 (9)
C4	0.0598 (13)	0.0760 (15)	0.146 (2)	−0.0169 (11)	0.0156 (15)	0.0058 (15)
C7	0.0235 (7)	0.0362 (7)	0.0274 (7)	−0.0005 (5)	−0.0044 (5)	−0.0015 (5)
C12	0.0244 (7)	0.0295 (6)	0.0296 (7)	−0.0005 (5)	0.0010 (5)	−0.0004 (5)
C13	0.0259 (7)	0.0350 (7)	0.0307 (7)	−0.0020 (5)	0.0008 (6)	−0.0019 (6)
C10	0.0293 (7)	0.0508 (9)	0.0295 (7)	0.0009 (6)	−0.0083 (6)	0.0011 (6)
C9	0.0326 (7)	0.0384 (7)	0.0261 (7)	−0.0026 (6)	0.0008 (6)	0.0001 (5)
C8	0.0222 (6)	0.0381 (7)	0.0307 (7)	−0.0027 (5)	0.0005 (6)	−0.0015 (6)
C11	0.0207 (6)	0.0447 (8)	0.0372 (8)	0.0010 (6)	−0.0014 (6)	0.0007 (6)
O1	0.0295 (6)	0.0745 (8)	0.0414 (6)	0.0125 (5)	0.0099 (5)	0.0059 (5)
O2	0.0382 (6)	0.0515 (6)	0.0290 (5)	0.0071 (5)	0.0002 (4)	0.0011 (4)
O3	0.0392 (6)	0.0692 (7)	0.0251 (5)	0.0010 (5)	0.0019 (4)	0.0036 (5)
O4	0.0215 (5)	0.0881 (9)	0.0340 (6)	−0.0020 (5)	0.0028 (4)	−0.0031 (6)
N1	0.0448 (8)	0.0357 (7)	0.0756 (10)	−0.0022 (6)	−0.0041 (7)	0.0009 (7)

### Geometric parameters (Å, °)

**Table:**

C6—C5	1.496 (3)	C4—H4B	0.9600
C6—H6A	0.9600	C4—H4C	0.9600
C6—H6B	0.9600	C7—C8	1.3795 (18)
C6—H6C	0.9600	C7—C12	1.3979 (18)
C1—N1	1.490 (2)	C7—H7	0.9300
C1—C2	1.511 (3)	C12—C11	1.386 (2)
C1—H1A	0.9700	C12—C13	1.4968 (19)
C1—H1B	0.9700	C13—O1	1.2476 (17)
C2—H2A	0.9600	C13—O2	1.2705 (16)
C2—H2B	0.9600	C10—C9	1.384 (2)
C2—H2C	0.9600	C10—C11	1.385 (2)
C3—N1	1.499 (2)	C10—H10	0.9300
C3—C4	1.517 (3)	C9—O3	1.3606 (17)
C3—H3A	0.9700	C9—C8	1.4039 (19)
C3—H3B	0.9700	C8—O4	1.3551 (16)
C5—N1	1.518 (3)	C11—H11	0.9300
C5—H5A	0.9700	O3—H3	0.8200
C5—H5B	0.9700	O4—H4	0.8200
C4—H4A	0.9600	N1—H12	0.916 (15)
			
C5—C6—H6A	109.5	C3—C4—H4C	109.5
C5—C6—H6B	109.5	H4A—C4—H4C	109.5
H6A—C6—H6B	109.5	H4B—C4—H4C	109.5
C5—C6—H6C	109.5	C8—C7—C12	121.57 (12)
H6A—C6—H6C	109.5	C8—C7—H7	119.2
H6B—C6—H6C	109.5	C12—C7—H7	119.2
N1—C1—C2	112.85 (16)	C11—C12—C7	118.75 (12)
N1—C1—H1A	109.0	C11—C12—C13	121.11 (12)
C2—C1—H1A	109.0	C7—C12—C13	120.12 (12)
N1—C1—H1B	109.0	O1—C13—O2	124.15 (13)
C2—C1—H1B	109.0	O1—C13—C12	118.18 (12)
H1A—C1—H1B	107.8	O2—C13—C12	117.67 (11)
C1—C2—H2A	109.5	C9—C10—C11	120.88 (13)
C1—C2—H2B	109.5	C9—C10—H10	119.6
H2A—C2—H2B	109.5	C11—C10—H10	119.6
C1—C2—H2C	109.5	O3—C9—C10	122.90 (12)
H2A—C2—H2C	109.5	O3—C9—C8	117.53 (12)
H2B—C2—H2C	109.5	C10—C9—C8	119.56 (13)
N1—C3—C4	114.51 (16)	O4—C8—C7	123.47 (12)
N1—C3—H3A	108.6	O4—C8—C9	117.53 (12)
C4—C3—H3A	108.6	C7—C8—C9	119.00 (12)
N1—C3—H3B	108.6	C10—C11—C12	120.22 (12)
C4—C3—H3B	108.6	C10—C11—H11	119.9
H3A—C3—H3B	107.6	C12—C11—H11	119.9
C6—C5—N1	113.88 (16)	C9—O3—H3	109.5
C6—C5—H5A	108.8	C8—O4—H4	109.5
N1—C5—H5A	108.8	C1—N1—C3	114.97 (15)
C6—C5—H5B	108.8	C1—N1—C5	111.29 (14)
N1—C5—H5B	108.8	C3—N1—C5	110.78 (14)
H5A—C5—H5B	107.7	C1—N1—H12	105.0 (13)
C3—C4—H4A	109.5	C3—N1—H12	106.2 (13)
C3—C4—H4B	109.5	C5—N1—H12	108.1 (12)
H4A—C4—H4B	109.5		

### Hydrogen-bond geometry (Å, °)

**Table:**

*D*—H···*A*	*D*—H	H···*A*	*D*···*A*	*D*—H···*A*
O3—H3···O2^i^	0.82	1.85	2.6574 (14)	166
O4—H4···O1^ii^	0.82	1.81	2.6321 (15)	178
C7—H7···O1^ii^	0.93	2.57	3.235 (2)	128
N1—H12···O2^iii^	0.92	1.87	2.776 (2)	170
C1—H1B···O3^iv^	0.97	2.57	3.409 (2)	145
C3—H3A···O1^v^	0.97	2.55	3.516 (2)	177
C3—H3B···O3^vi^	0.97	2.56	3.351 (2)	139
C10—H10···O4^vii^	0.93	2.38	3.222 (2)	150

Symmetry codes: (i) *x*, −*y*+1/2, *z*+1/2; (ii) *x*−1/2, −*y*+1/2, −*z*+1; (iii) *x*−1/2, −*y*+3/2, −*z*+1; (iv) −*x*+1, *y*+1/2, −*z*+3/2; (v) *x*−1, *y*+1, *z*; (vi) *x*−1/2, *y*+1, −*z*+3/2; (vii) *x*+1/2, *y*, −*z*+3/2.
